# Evaluation of implementation of evidence-based public health training in sub-Saharan Africa

**DOI:** 10.4102/jphia.v15i1.576

**Published:** 2024-08-30

**Authors:** Anke C. Rohwer, Nasreen S. Jessani, Nyanyiwe M. Mbeye, Bonny E. Balugaba, Ann R. Akiteng, David Tumusiime, Seleman Ntawuyirushintege, Kiya Kedir, Rawleigh Howe, Solange Durao, Ingrid Toews, Jacob Burns

**Affiliations:** 1 Centre for Evidence-based Health Care, Division of Epidemiology and Biostatistics, Department of Global Health, Faculty of Medicine and Health Sciences, Stellenbosch University, Cape Town, South Africa; 2 Institute of Development Studies, Brighton, United Kingdom; 3 Department of Community and Environmental Health, School of Global and Public Health, Kamuzu University of Health Sciences, Blantyre, Malawi; 4 Department of Disease Control and Environmental Health, School of Public Health, College of Health Sciences, Makerere University, Kampala, Uganda; 5 College of Health Sciences, Makerere University, Kampala, Uganda; 6 College of Medicine and Health Sciences, University of Rwanda, Kigali, Rwanda; 7 Armauer Hansen Research Institute, Non-Communicable Disease Ministry of Health, Addis Ababa, Ethiopia; 8 Health Systems Research Unit, South African Medical Research Council, Cape Town, South Africa; 9 Institute for Evidence in Medicine, Medical Center, Faculty of Medicine, University of Freiburg, Freiburg, Germany; 10 Institute for Medical Information Processing, Biometry, and Epidemiology, LMU Munich, Munich, Germany

**Keywords:** evidence-based public health, RE-AIM framework, PRISM, evidence-informed decision-making, capacity building, Africa

## Abstract

**Background:**

The Collaboration for Evidence-based Healthcare and Public Health in Africa (CEBHA+) developed and offered a course on evidence-based public health (EBPH) in five sub-Saharan African (SSA) countries to enhance individual and institutional capacity.

**Aim:**

This study aims to assess, compare and learn from implementing the CEBHA+ EBPH course using the Reach, Effectiveness, Adoption, Implementation, Maintenance (RE-AIM) framework and Practical, Robust, Implementation and Sustainability Model (PRISM).

**Setting:**

This study involved CEHBA+ partner universities in five countries in SSA.

**Methods:**

We developed a framework that draws on signalling questions for RE-AIM and PRISM dimensions. Country teams reflected on, discussed and mapped unique experiences. Using this framework, we then elicited common themes across countries and distilled country-specific experiences through virtual discussions.

**Results:**

Across countries, 130 public health practitioners, researchers and students completed the course (Reach). The course increased EBPH knowledge and skills and the capacity to teach EBPH and resulted in immediate opportunities for applying skills (Effectiveness). Hybrid offering in two countries presented challenges regarding Internet connectivity and hybrid discussions. Facilitators had previous training in teaching EBPH. While learning material was the same across countries, the content was adapted to represent local public health priorities (Implementation, Adoption). Course materials have informed other related training leading to spin-offs (Maintenance). Institutionalisation is dependent on external funding.

**Conclusion:**

Strengthening EBPH capacity across contexts is feasible. Curricula containing both core and contextualised elements create an authentic learning environment. Formal evaluations should be embedded within capacity-strengthening initiatives.

**Contribution:**

This is the first study evaluating EBPH training in SSA using an implementation science lens, offering learning about context-relevant adaptations that assist with plans for sustainability and scale.

## Introduction

Evidence-based public health (EBPH) is defined as:

[*T*]he conscientious, explicit and judicious use of current best evidence in making decisions about the care of communities and populations in the domain of health protection, disease prevention, health maintenance and improvement (health promotion).^1^

Decisions about public health interventions should therefore be informed by the best available evidence, considerations from various stakeholders, preferences and values of communities, available resources, cost-effectiveness and other factors.^2^ This is particularly important in contexts with high burden of disease compounded by scarce resources to address them.^3,4^

Multiple public health emergencies such as human immunodeficiency virus (HIV), malnutrition, the rise of non-communicable diseases and, most recently, coronavirus disease 2019 (COVID-19) highlight the importance of making evidence-informed public health decisions.^5,6^ It is therefore essential that public health practitioners, decision-makers, guideline development groups and researchers have the necessary knowledge and skills to find, appraise and interpret evidence on public health interventions.^4^ However, our targeted and systematic searches in 2018 for existing training initiatives focusing on EBPH in sub-Saharan Africa (SSA) suggested that these are lacking.^7^

Building long-term capacity in evidence-based healthcare and EBPH was a critical objective of the Collaboration for Evidence-based Healthcare and Public Health in Africa (CEBHA+), a 5-year project (2017–2022) funded by the German Ministry of Education and Research (BMBF). It comprised seven African partners in Rwanda, Uganda, Malawi, Ethiopia and South Africa and two German partners.

Within CEBHA+, capacity development was conceptualised at the individual, institutional and system level. In response to the limited training initiatives in the field of EBPH in SSA and the rest of the world, the CEBHA+ Capacity Development Working Group developed a course and accompanying training material in 2018 to enhance individual as well as institutional capacity on EBPH.

The CEBHA+ EBPH course aimed to introduce the concepts of EBPH and focused on formulating clear questions on finding, appraising, interpreting and applying the best evidence about public health questions relevant to the setting within each country. It was implemented as a 5-day workshop in each of the African CEBHA+ countries. Specific learning outcomes and topics covered per day are summarised in Table 1.^8^

**TABLE 1:** Learning outcomes for the Collaboration for Evidence-based Healthcare and Public Health in Africa’s evidence-based public health workshop.

Day	Topic	Learning outcomes *After the workshop, participants should be able to:*
Day 1	Introduction to EBPH	Explain and discuss the principles and importance of EBPH Explain concepts of ‘complex interventions’ and ‘complex systems’ and understand how these affect the evaluation of public health interventions in primary research and evidence synthesis
	EBPH step 1: Phrasing questions	Formulate clear questions relevant to public health in Africa
Day 2	EBPH step 2: Searching for evidence	Search for the best available evidence to answer the question
Day 3	EBPH step 3: Reading primary studies relevant to public health	Describe common study designs used to evaluate public health interventions Critically appraise a controlled before-after study on a public health intervention and interpret results
Day 4	EBPH step 3: Reading systematic reviews	Describe the value of research synthesis in public health Critically appraise systematic reviews and interpret results
Day 5	EBPH step 4: Interpreting and applying results	Discuss the applicability of evidence to make evidence-informed decisions in a public health context Outline the value of public health guidelines and discuss concepts of evidence use in policy and practice

Note: The steps of EBPH, as portrayed in the Topic column, are adapted from Sackett et al. 2000.

EBPH, evidence-based public health.

The CEBHA+ EBPH course was first offered in Kampala, Uganda, in October 2018, with subsequent courses in Kigali, Rwanda (September 2019); Blantyre, Malawi (August 2021); Addis Ababa, Ethiopia (December 2021) and Cape Town, South Africa (October 2022). The target audience was public health practitioners, researchers and students, while presenters comprised external and local facilitators. The interactive learning approach included didactic input, scenarios, small group discussions and exercises and other active learning strategies.

All participants received a workbook containing the relevant readings and exercises used during the course, while additional resources and presentations were shared via an online classroom. During the first workshop in Uganda, we recognised that a tangible summary of what was covered during the week would be useful. We therefore developed an EBPH Pocket guide^9^ to complement the didactic input and distributed this to workshop participants in Rwanda, Malawi, Ethiopia and South Africa.

Even though the content and material were the same for all workshops, various internal and external factors influenced the implementation of the training. This study aims to assess, compare and learn from the implementation of the CEBHA+ EBPH course in five different countries using the Reach, Effectiveness, Adoption, Implementation, Maintenance (RE-AIM) framework combined with the Practical, Robust, Implementation and Sustainability Model (PRISM).

## Research methods and design

### Study design

We conducted a retrospective qualitative evaluation of the EBPH course using a systematic approach underpinned by the RE-AIM framework with some aspects from the PRISM framework. Specifically, using these frameworks, for each country, we first reflected on and described our experiences in developing and implementing the EBPH workshop. Next, we compared experiences across countries, focusing on summarising common, diverging and country-specific themes.

### Data collection and analysis

Several frameworks exist to evaluate the implementation of interventions and to understand factors influencing implementation. The RE-AIM framework was initially developed to evaluate public health interventions^10,11^ and has been widely used to plan and evaluate interventions across a variety of fields, including education.^12^ It consists of five implementation dimensions, which the framework defines as follows: *Reach* refers to the absolute proportion, number and representativeness of participants; *Effectiveness* refers to the impact of the intervention on important outcomes; *Adoption* refers to the absolute number of settings and intervention agents willing to initiate a programme; *Implementation* refers to the fidelity and consistency of implementing an intervention, as well as adaptations made; and *Maintenance* refers to the extent to which a programme becomes institutionalised.^9^ Given that RE-AIM assesses outcomes of the intervention both at individual and organisational level, emphasising both internal and external validity and transparency, it was deemed suitable to evaluate the implementation of the CEBHA+ EBPH course. The Practical, Robust, Implementation and Sustainability Model (PRISM), in its entirety, aims to support the translation of research into practice and comprises multiple dimensions. In our approach, we aimed to complement the RE-AIM framework by using the parts of the PRISM model focusing on describing the *internal* and *external contextual factors* that influence the outcomes of an intervention.^13^ We therefore used PRISM to capture the multi-level contextual influences of the RE-AIM outcomes.

As a first step, three authors (A.C.R., N.S.J. and J.B.) clarified and discussed each of the RE-AIM and PRISM dimensions to ensure a mutual understanding of the issues they addressed. Based on existing definitions and descriptions of the dimensions in the literature, the three authors expanded on these by adding signalling questions specifically related to the CEBHA+ EBPH course, which were organised in a template (Online Appendix 1).

As a second step, guided by these signalling questions, the team responsible for implementing the course in each country met to reflect on and discuss the implementation of the CEBHA+ EBPH course in their respective countries. Throughout this process, they considered the experiences of local and external facilitators and drew on participant feedback. Each team mapped their experiences onto the template created, resulting in a spreadsheet with descriptions of the RE-AIM and PRISM dimensions for all five countries.

In the third step, the author team met virtually to discuss and compare experiences across countries as recorded on the spreadsheet. This in-depth, cross-country reflection involved systematically reviewing each dimension to elicit common themes and distil country-specific experiences. In the final step, we integrated contextual factors (PRISM) within each of the RE-AIM domains.

### Ethical considerations

We did not seek ethical approval for this study, as we did not include any participant data in our report. Our findings are based on reflections and experiences of the author team.

## Results

**FIGURE 1:**
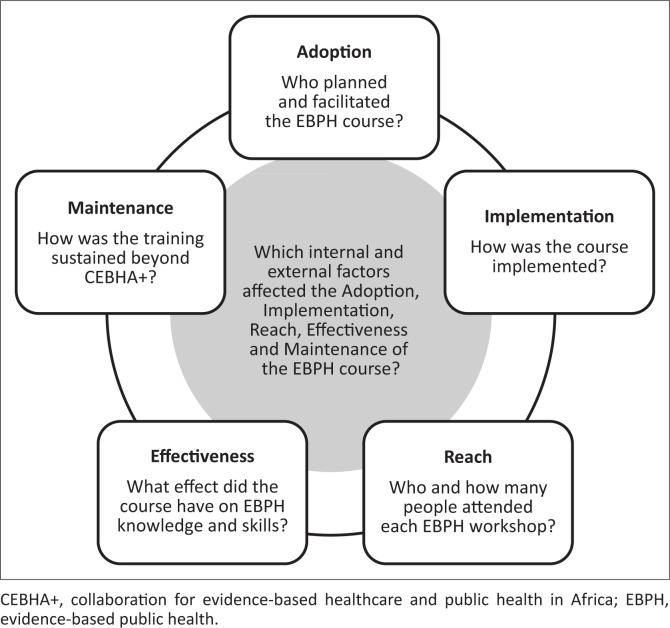
Reach, Effectiveness, Adoption, Implementation, Maintenance and Practical, Robust, Implementation and Sustainability Model dimensions to evaluate the evidence-based public health course, including the overarching questions that were addressed within each dimension.

The detailed findings per country and RE-AIM and PRISM domains are available in Online Appendix 1. Synthesised findings are summarised below, not in the RE-AIM order, but in the order that we felt best captured the repeated and iterative offering of the workshop: Adoption, Implementation, Reach, Effectiveness, Maintenance. Figure 1 outlines the assessed dimensions and the overarching questions addressed as part of each dimension.

### Adoption

Capacity building was one of four cross-cutting work packages of the BMBF-funded CEBHA+ project. The EBPH course was developed in response to an identified capacity gap, a recognised need by partners and a key deliverable for the project. Each CEBHA+ partner institution nominated one member to join the project’s Capacity Development Working Group to implement activities including, among other activities, the EBPH course. These members had diverse backgrounds and expertise, and importantly, many had limited experience in teaching EBPH. Strengthening the capacity of CEBHA+ partners to *teach* EBPH was therefore embedded within the implementation of the EBPH course.

Two expert facilitators (A.C.R. and J.B.) led the development of the EBPH course and related learning material, with input from the CEBHA+ Capacity Building Working Group. Novice and experienced facilitators from the CEBHA+ partner countries underwent formal training in teaching evidence-based healthcare (EBHC) and attended the semester-long teaching EBHC short course at Stellenbosch University in 2019. This helped them to adopt and adapt the material for their own contexts and to facilitate interactive learning activities as part of the course.

A.C.R. and J.B. contributed to all five EBPH workshops in terms of planning, adapting, implementing and facilitating. Originally, the plan was for all facilitators to partake in experiential learning as a capacity-strengthening endeavour for teaching EBHC. Collaboration for Evidence-based Healthcare and Public Health in Africa facilitators that attended the teaching EBHC course joined an EBPH workshop in another country to observe and assist with planning and facilitation, prior to hosting an EBPH workshop in their home country, where they were more actively involved in facilitating sessions.

Experienced facilitators provided guidance and mentorship, and the team reflected on sessions throughout this process. Because of the COVID-19 pandemic, Ethiopian facilitators were unable to attend an EBPH workshop before hosting their own. However, they were supported virtually by CEBHA+ facilitators and joined by facilitators from the Knowledge Management Directorate at Armauer Hansen Research Institute (AHRI) and the Ethiopian Public Health Institute who have prior experience of delivering similar courses. In South Africa, all facilitators had experience in teaching EBHC and related topics.

### Implementation

For each workshop, we adapted the materials to align with the local context. Specifically, these included a presentation on the local burden of disease and public health context specific to each country, selecting studies addressing local public health problems for course exercises and showcasing local experiences of using evidence in policy and practice. Apart from this, the workshops were implemented in a largely uniform manner. We held a short evaluation at the end of each day to identify issues in the implementation that could be readily addressed for the remainder of the workshop. These included timing of sessions, logistics and format of course exercises.

The biggest adaptation in the implementation of the course was the hybrid offerings in Malawi and Ethiopia, where the workshops were implemented during the COVID-19 pandemic, when international travel restrictions were in place. Participants were nevertheless able to join in-person, and although external facilitators were able to join the workshops virtually to present on certain topics, limited Internet connectivity was a challenge in both countries. We tried to overcome this by recording presentations and making these available to workshop participants; however, we were unable to facilitate rich discussions between external presenters and workshop participants. Furthermore, external facilitators could not join the exercises, which were closely linked to the didactic input. There might have thus been some disconnect between didactic input and the application of knowledge and skills.

Local institutions supported CEBHA+ teams to host and offer the workshops, including providing a venue or assisting with procuring one, advertising the course, using institutional processes to manage travel and accommodation where necessary, printing and providing Internet access and information technology (IT) support. Key to the success of the course was CEBHA+ funding that funded facilitators’ time and travel, venues, participants’ registration fees, catering and printing of workshop materials. Administrative staff at the respective institutions assisted country teams with logistic issues related to the implementation of the course. For example, in South Africa and Ethiopia, administrators had to arrange travel for international and national participants at short notice and therefore played a key role in the successful delivery of the course. In other countries, administrators assisted with travel arrangements for facilitators, including Visa applications, accommodation and local transport.

### Reach

Workshops in each country were advertised widely. We aimed to include a diverse range of participants that could benefit from the course, including public health practitioners, researchers and students. Within these categories, we also aimed to ensure diversity related to factors such as age, sex and level of expertise. As spaces were limited to 30 participants per workshop, interested participants applied to attend by completing a short application form on Google forms. Collaboration for Evidence-based Healthcare and Public Health in Africa covered all expenses related to the course, including registration fees, ensuring that cost would not be a deterrent for those who applied. In Ethiopia and South Africa, CEBHA+ was able to cover travel and accommodation for some participants.

Overall, many people were interested in attending the course, with 104 applications in Uganda, 130 in Rwanda, 114 in Malawi, 56 in Ethiopia and 42 in South Africa. The workshop facilitation teams selected applicants based on previous training in epidemiology and biostatistics and their motivation to attend the course. Furthermore, we tried to ensure that our target population, as described above, was adequately represented. Motivations to attend the course included to gain knowledge and skills in EBPH and systematic reviews so that these could be applied to their current work, to expand their knowledge on public health decision-making, to use the skills in ongoing systematic reviews, to understand how to apply global evidence in a local context, to gain skills linked to a PhD and to become a champion for evidence-informed decision-making and knowledge translation.

In Ethiopia, we had much more male compared to female applicants. We thus included all women who met the minimum inclusion criteria to ensure that they were adequately represented in the final cohort. In South Africa, all applicants were invited to participate given the lower number of applications received. However, more than a third of applicants were from outside of South Africa, and those who were unable to fund travel or obtain a Visa did not attend. In Malawi, the workshop was initially planned for May 2020, and participants were selected before the start of the pandemic. Because of several pandemic-related postponements, some applicants had other commitments by the time the workshop was implemented and were no longer able to attend.

In most cases, successful applicants proceeded to attend the course. However, some declined to participate mainly because of a lack of funding to support travel or not being able to commit to attending five full days. Where selected applicants declined to participate, we invited applicants from the waiting list.

A total of 130 participants attended the EBPH workshops in Uganda (*n* = 30), Rwanda (*n* = 30), Malawi (*n* = 22), Ethiopia (*n* = 26) and South Africa (*n* = 22). Within each country, we were able to successfully recruit a participant pool reflecting our target population. Participants had diverse backgrounds and various levels of pre-existing knowledge on systematic reviews and EBPH. Some participants had already conducted systematic reviews, were involved in conducting reviews at the time of the workshop or had attended previous training in systematic reviews and evidence-informed decision-making. In South Africa, some participants had completed a Master of Science in Clinical Epidemiology at Stellenbosch University. However, across countries, most participants had limited background knowledge in evidence-informed decision-making.

### Effectiveness

In all countries, participants actively engaged with the interactive exercises to apply the knowledge that they had learned and had lively discussions within their small groups. Prior to the course, some participants had limited knowledge in EBPH, but these reported gaining new knowledge and skills because of attending the training. Participants who had some previous experience with systematic reviews and/or evidence-based healthcare indicated that the workshop consolidated what they had previously learned and provided an opportunity to deepen their understanding of certain concepts. Across countries, there was evidence that skills learned during the workshop were applied. For example, participants requested input into ongoing research projects or systematic reviews. In Uganda, one participant became a co-author of a systematic review undertaken as part of the CEBHA+ research agenda, while two participants who attended the initial workshop in 2018 were facilitators of the second workshop. Taken together, these aspects do suggest that the EBPH workshop had a positive effect.

### Maintenance

The CEBHA+ EBPH course was registered as a short course at Stellenbosch University, allowing participants in all countries to receive a certificate of attendance. Initially, we had planned for the course to become a formal module, embedded in the programmatic offering at respective institutions of African CEBHA+ partners. Although all institutions were supportive of and enthusiastic about offering the EBPH course, to date, it has only been offered as a stand-alone short course. Furthermore, it is still dependent on external funding to cover expenses related to facilitators’ time, learning materials, venues and participants’ registration fees.

Demand for the course was high, and in some cases, it led to additional workshop offerings or ‘spin-off’ offerings. For instance, Ugandan project partners offered a second workshop in 2019; those in Rwanda developed a series of lunchtime sessions based on the content of the EBPH course for students, researchers and faculty members and in Ethiopia, members of ‘Fenot’, a non-governmental organisation of public health advisors, and the Ethiopian Public Health Institute have already eagerly integrated the EBPH course into their existing training programmes.

It is not clear whether the EBPH course in its current form will be maintained beyond the CEBHA+ funding period.

## Discussion

Between 2018 and 2022, the CEBHA+ network developed and implemented a 5-day, in-person course on EBPH to strengthen capacity in evidence-informed decision-making. The course was offered in five African countries: Uganda, Rwanda, Malawi, Ethiopia and South Africa. We assessed and reflected on the implementation of the course across the different settings using the RE-AIM and PRISM framework.

### Reflections and lessons learned

Engagement with local facilitators throughout the process facilitated *Adoption*, local adaptation and *implementation* of the course. This approach also supported capacity strengthening for teaching EBPH and EBHC, with the opportunity for those staff members from CEBHA+ partners with less experience with the topic to become involved in an iterative manner. This also increases the pool of facilitators who could conduct such training in the future. Although offering the EBPH course was a grant requirement for all African CEBHA+ partners, everyone was highly motivated to offer the course in their context. Collaboration and cooperation between CEBHA+ partners were key in successfully offering the workshops. Involvement of the two senior facilitators in all the workshops helped to ensure continuity and fidelity.

In reflecting on the lessons learned for the *implementation*, we feel it was imperative to adapt and tailor the content for the local context for a more authentic and engaging learning environment. Facilitators with different professional backgrounds and levels of EBPH experience helped to ensure wide-ranging discussions. Daily evaluations were a helpful instrument for receiving feedback that could help improve the implementation for the remainder of the workshops. The emergence of the COVID-19 pandemic led to offering of hybrid workshops, in which the participants attended in-person, while some external facilitators attended online. This proved that, while virtual learning was possible, certain practical aspects simply worked better in-person. More creative means to ensure the same impact with virtual or hybrid offerings need to be considered.

We maintain that there was clearly a high demand for this course in all CEBHA+ partner countries from local as well as international participants, thereby indicating great *Reach*, interest and the potential for sustainability. The number and diversity of applicants across countries suggest that context-specific internal factors such as strategies for advertising and recruiting, as well as external factors such as timing and windows of opportunity, are likely to affect the reach. The fact that CEBHA+ was able to cover registration fees for the course as well as travel and accommodation for some likely positively influenced this reach, suggesting that such mechanisms may be important in this context.

Related to *Effectiveness*, we learned that the baseline knowledge and experiences varied broadly across participants. Ensuring a balance of researchers, public health practitioners and students led to rich discussions shaped by varying backgrounds and experiences. This meant that, if all participants were to benefit, the course structure and content needed to be developed in a way that recognised, reflected and accommodated this. Iterative training and teaching for facilitators less experienced in teaching EBPH proved to be an effective way to incorporate new facilitators into the team. Taken together, these aspects do suggest that the EBPH workshop had a positive effect on capacity; however, the current evaluation strategy does not allow us to draw firm conclusions. While informal feedback from participants clarified value and effectiveness, formalised evaluations from participants and a more formal evaluation of the effect of the course in the future would allow for a more systematic and rigorous capture of participant experiences as well as guide improved design and implementation of the course.

We felt that offering the course as a short course with a certificate from a recognised university was appreciated by participants and represented an additional incentive for completing the course. This was a significant learning under the *Maintenance* domain. For the long-term sustainment of the EBPH course, although the interest and demand exist at all partner institutions, the transition from an externally funded, time-limited workshop to a course embedded within local curricula is challenging and remains unresolved. Factors influencing this transition include tedious institutional accreditation processes, willingness to take ownership of a new module and divergent priorities of externally funded faculty members.

### Comparisons to evaluations of other training

The teaching of evidence-based practice has been examined in the literature, including general evaluations of teaching the topic,^14^ evaluations of specific modules^15,16^ and assessments of existing tools for evaluation.^17^ However, we are not aware of any studies evaluating the implementation of an EBPH course in the African context.

Given ‘fit’ and ‘readiness’ were already established, a framework such as the Context Compass framework^18^ would not have assisted with the evaluation of the intervention. The RE-AIM and PRISM frameworks, however, helped to capture our reflections and to compare the implementation of the EBPH workshop across settings – similar to its use for other educational interventions.^19,20,21,22^

A deeper understanding of the various actors (within our sphere of intervention) involved and the external domains (within as well as outside our sphere of influence) might have been helpful in the contextual assessment. While the Consolidated Framework for Implementation Research (CFIR)^23^ does impress upon these, we applied the PRISM alongside RE-AIM to reflect on the inner and outer contexts that affected the implementation of the workshops.

### Limitations

While a more formal, prospective evaluation, which, for example, uses mixed-methods to explore the effect of the EBPH course both qualitatively and quantitatively, may have been more informative, we did not have the resources to do so. Instead, we based our findings on reflections and experiences of country teams offering the course. Multiple steps in our applied methodology, including the country teams’ collection and description of their experiences, as well as the work of the three authors (A.C.R., N.S.J. and J.B.) in compiling and summarising these experiences, are shaped by our individual and shared experiences. However, apart from these limitations, we feel there is clear value in sharing these experiences from the perspective of those planning and implementing such a workshop. A.R. and J.B. participated in the planning and offering of all the workshops and were therefore able to not only reflect on individual workshops but also see the bigger picture and compare offerings of the course in the various countries.

Furthermore, we did not include the perspectives of workshop participants. Although we collected their informal feedback through daily evaluation forms, we did not seek ethics approval to use these data for research purposes. Our assessment of the effectiveness of the EBPH course was limited, as we did not collect objective data on EBPH knowledge and skills pre- and post-workshops. Using a validated tool to assess change in knowledge and skills and interviewing participants on their experience with the course would add valuable insights that could complement our findings.

## Conclusion

Throughout the development and implementation of an EBPH course in five SSA countries, we gained valuable experience and learned several lessons, which we feel are highly relevant for others planning to undertake similar initiatives. Strengthening capacity in evidence-informed decision-making and EBPH across contexts is possible and valuable. However, it is important to create curricula that contain both core elements and flexible, contextualised elements to ensure an authentic learning environment. Increasing the pool of EBPH teachers is a sustainable way to ensure that EBPH training is maintained beyond the project period; however, wide reach and maintenance may depend on local or external funding to ensure that training is low cost and thus available to a diverse population. Such training initiatives should embed research and formal evaluations, which could be prospectively planned and utilise mixed-methods approaches.
